# La trayectoria de un hipnotista en Buenos Aires como espejo de las transformaciones sanitarias (y) globales de fines del siglo XIX

**DOI:** 10.1590/S0104-59702023000100013

**Published:** 2023-04-03

**Authors:** José Ignacio Allevi

**Affiliations:** i Investigador asistente, Instituto de Humanidades y Ciencias Sociales del Litoral/CONICET-Universidad Nacional del Litoral. Santa Fe – Santa Fe – Argentina; ii Profesor asistente, Facultad de Psicología/Universidad Nacional de Rosario. Rosario – Santa Fe – Argentina. joseignacio.allevi@gmail.com

Si acaso podemos afirmar que la historiografía de la salud, la enfermedad y la medicina presenta en la última década nuevas preguntas y desafíos que apuntalan su renovación, esta obra resulta un claro ejemplo de estos últimos. La detallada investigación que Vallejo (2021) emprende sobre la breve trayectoria de Alberto Díaz de la Quintana en Buenos Aires, entre 1886 y 1895, le permite articular con erudita pericia una serie de procesos de alcance diverso, localizados en una ciudad por ellos transformada. Aunque elija no presentar su trabajo bajo este rótulo, podría afirmarse que, tanto por su rigurosa reconstrucción de las dinámicas que transformaron el mundo durante el siglo XIX (Osterhammel, 2014) como por las preguntas que hace sobre su objeto de indagación, la pesquisa que nutre este libro constituye un ejercicio historiográfico global (Jackson, 2018).

Valiéndose de las iniciativas de este hipnotista como excusa, Vallejo se aboca al estudio de una trama de actores, objetos y prácticas a cuyo estudio la historiografía local no se ha abocado, o lo ha hecho mayormente siguiendo la documentación estatal o médica. En efecto, su trabajo le permite iluminar las tensiones que atravesaban al vasto campo de actores que ofrecían sus servicios para el restablecimiento de la salud, así como las que signaban al Estado y los médicos argentinos para controlar su experticia. En su afán, logra exponer no solo el impacto que sobre estos últimos tuvo la revolución microbiana durante el último tercio del siglo, sino también el ambiguo rol del Departamento Nacional de Higiene que, en la mayoría de los casos, en vez de velar por las demandas de la corporación médica, funcionó como síntoma de su falta de consensos.

Una manera de entender por qué los hipnotistas podían desempeñarse de facto en la Buenos Aires *fin de siécle* se encuentra, así, en las disputas al interior de una experticia en vías de profesionalización. Sin embargo, Vallejo ilumina al menos dos factores clave, y poco explorados hasta el momento, para entender por qué los mismos diplomados terminaban por valerse de recursos y estrategias no autorizados por su propia corporación: el dinero y la creciente circulación global de objetos terapéuticos ofrecidos al consumo masivo, con escasa regulación oficial. Ello respondía no solo a sus limitaciones materiales y al desconocimiento al respecto, sino también al hecho de que muchos médicos participaban de su comercialización.

En esta dirección, el escenario de la medicina “oficial” y “popular” que Vallejo logra delinear da cuenta de la dificultad para catalogar a sus actores bajo el binarismo de médicos o curanderos/charlatanes. En palabras del autor, “se trataba más bien de campos con límites difusos, y zonas híbridas donde la proliferación de identidades impedía el uso de rótulos perennes” (Vallejo, 2021, p.27).

En relación a las fuentes utilizadas, y a diferencia de la historiografía local (e incluso de sus propios trabajos), el rasgo distintivo del nuevo libro de Vallejo reside en auscultar sus preguntas de manera cuidada y minuciosa sobre la prensa periódica general, nacional y extranjera, junto con algunos documentos “oficiales” a los cuales logra interrogar desde sus propios márgenes. Su elección porta una ventaja innegable, enunciada por su autor, a saber, “que esas páginas guardan testimonio de múltiples actores, mediaciones, artefactos y procesos que fueron constitutivos del desarrollo efectivo del mundo de la salud, pero de los cuales no quedan casi rastros en otros soportes documentales” (Vallejo, 2021, p.34). Podría objetarse que el uso “obstinado” de esta fuente acarrea una limitación sobre el mundo que puede iluminar, urbano y letrado. Aun cuando los índices de alfabetización argentinos guardaban un largo trayecto por delante, Vallejo da cuenta de la especificidad de Buenos Aires en este sentido, documentando la creciente expansión de la prensa en comparación con otras grandes urbes del período.

En su hermenéutica, Vallejo logra una combinación poco habitual entre rigurosidad analítica con una narrativa sagaz y llevadera de una miríada de acontecimientos sobre la escena cultural y sanitaria porteña que, de no mediar la acción documentalista y metódica del autor, podrían pensarse como ficcionales. Entre ellos, el autor recupera el desempeño de su hipnotista en Madrid, su circulación por la prensa de La Habana y Manila, hasta su desembarco en Buenos Aires. Allí, en poco tiempo alcanzó una difusión notable por contar con el beneplácito de la comunidad española, al que luego se sumaron otros de distinta envergadura. Por encima de las acusaciones y denuncias que debió enfrentar, si algo queda fuera de duda en esta investigación es la capacidad de Díaz de la Quintana para manejar tales conflictos desde su condición de publicista y de la espectacularización de sus servicios, de la mano de una sonámbula que lo acompañaba desde Madrid.

En esta dirección, esta trayectoria singular y excéntrica le permite al autor exhibir con soltura una serie de aspectos del mundo sanitario porteño de fin de siglo. En particular, merece atención su revalorización de la prensa como ámbito para auscultar la oferta y circulación de una variedad notable de servicios y objetos para el restablecimiento de la salud, pero también como ámbito de resolución de los conflictos que la corporación médica suscitaba para controlarlos. En igual medida, el libro es coherente con su estrategia metodológica al dar cuenta de que la respuesta estatal frente a las iniciativas de este hipnotista evidenciaba más tensiones internas a la corporación médica que unidad de acción. De manera más general, pero igualmente concreta, este libro recupera y reactualiza una pregunta trunca de la historiografía médica local de los años 1980, a saber, el lugar del componente inmigratorio en la conformación de una corporación médica local. En dicho gesto, abre una cantera de investigación que, de continuarse, arrojaría prolíficos resultados.

Un hallazgo en particular amerita ser destacado. Vallejo percibe con claridad que fueron estos personajes híbridos quienes identificaron y respondieron al tono de una subjetividad urbana ávida de objetos y prácticas terapéuticas para auto-intervenirse. Al igual que en otras capitales occidentales, este fenómeno fungía en Buenos Aires a espaldas de la comunidad médica. El autor logra así invertir de manera inteligente las tesis sobre la medicalización social para mostrar que, por el contrario, si actores como Díaz de la Quintana podían alimentar semejantes fantasías, ello era posible en razón a un público dispuesto a consumirlas.

## References

[B1] JACKSON Mark (2018). A global history of medicine.

[B2] OSTERHAMMEL Jürgen (2014). The transformation of the world: a global history of the nineteenth
century.

[B3] VALLEJO Mauro (2021). Hipnosis e impostura en Buenos Aires: de médicos, sonámbulas y
charlatanes a fines del siglo XIX.

